# Unexplained chronic liver disease in Ethiopia: a cross-sectional study

**DOI:** 10.1186/s12876-018-0755-5

**Published:** 2018-02-13

**Authors:** Stian Magnus Staurung Orlien, Nejib Yusuf Ismael, Tekabe Abdosh Ahmed, Nega Berhe, Trine Lauritzen, Borghild Roald, Robert David Goldin, Kathrine Stene-Johansen, Anne Margarita Dyrhol-Riise, Svein Gunnar Gundersen, Marsha Yvonne Morgan, Asgeir Johannessen

**Affiliations:** 1 Regional Centre for Imported and Tropical Diseases, Oslo University Hospital Ullevål, Oslo, Norway; 2 Department of Internal Medicine, Hiwot Fana Specialized University Hospital, Harar, Ethiopia; 3 Haramaya University College of Health and Medical Sciences, Harar, Ethiopia; 4 Department of Internal Medicine, Jugal Hospital, Harar, Ethiopia; 5 Aklilu Lemma Institute of Pathobiology, Addis Ababa University, Addis Ababa, Ethiopia; 6 Department of Medical Biochemistry, Vestre Viken Hospital Trust, Drammen, Norway; 7 Department of Pathology, Oslo University Hospital Ullevål, Oslo, Norway; 8 Institute of Clinical Medicine, Faculty of Medicine, Oslo University, Oslo, Norway; 9 Centre for Pathology, Imperial College London, London, UK; 10 Department of Molecular Biology, Norwegian Institute of Public Health, Oslo, Norway; 11 Department of Infectious Diseases, Oslo University Hospital Ullevål, Oslo, Norway; 12 Department of Clinical Science, University of Bergen, Bergen, Norway; 13 Research Unit, Sørlandet Hospital HF, Kristiansand, Norway; 14 Department of Global Development and Planning, University of Agder, Kristiansand, Norway; 15 UCL Institute for Liver & Digestive Health, Division of Medicine, University College London, Royal Free Campus, London, UK; 16 Department of Infectious Diseases, Vestfold Hospital Trust, Tønsberg, Norway

**Keywords:** Hepatotoxicity, Epidemiology, *Catha edulis*, Viral hepatitis, Sub-Saharan Africa

## Abstract

**Background:**

Hepatitis B virus (HBV) infection is assumed to be the major cause of chronic liver disease (CLD) in sub-Saharan Africa. The contribution of other aetiological causes of CLD is less well documented and hence opportunities to modulate other potential risk factors are being lost. The aims of this study were to explore the aetiological spectrum of CLD in eastern Ethiopia and to identify plausible underlying risk factors for its development.

**Methods:**

A cross-sectional study was undertaken between April 2015 and April 2016 in two public hospitals in Harar, eastern Ethiopia. The study population comprised of consenting adults with clinical and radiological evidence of chronic liver disease. The baseline evaluation included: (i) a semi-structured interview designed to obtain information about the ingestion of alcohol, herbal medicines and local recreational drugs such as khat (*Catha edulis*); (ii) clinical examination; (iii) extensive laboratory testing; and, (iv) abdominal ultrasonography.

**Results:**

One-hundred-and-fifty patients with CLD (men 72.0%; median age 30 [interquartile range 25–40] years) were included. CLD was attributed to chronic HBV infection in 55 (36.7%) individuals; other aetiological agents were identified in a further 12 (8.0%). No aetiological factors were identified in the remaining 83 (55.3%) patients. The overall prevalence of daily khat use was 78.0%, while alcohol abuse, defined as > 20 g/day in women and > 30 g/day in men, was rare (2.0%). Histological features of toxic liver injury were observed in a subset of patients with unexplained liver injury who underwent liver biopsy.

**Conclusion:**

The aetiology of CLD in eastern Ethiopia is largely unexplained. The widespread use of khat in the region, together with histopathological findings indicating toxic liver injury, suggests an association which warrants further investigation.

## Background

‘Chronic liver disease’ (CLD) is the term used to describe disordered liver function lasting for six or more months. It results from a process of progressive destruction and regeneration of the liver parenchyma and encompasses a wide range of liver pathologies including: chronic hepatitis, cirrhosis and hepatocellular carcinoma. CLD is a major cause of morbidity and mortality, and was responsible for an estimated 1.3 million deaths worldwide in 2015 [1]. The commonest causes of CLD are chronic infection with hepatitis B (HBV) or C (HCV), alcohol misuse and non-alcoholic fatty liver disease (NAFLD) [2].

Ethiopia is a low-income country in East Africa with a population of nearly 100 million [3]. The prevalence of CLD in Ethiopia is largely unknown but is assumed to be high [4]. The estimated seroprevalence of hepatitis B surface antigen (HBsAg) in Ethiopia is 6.0% [5] and of HCV-antibody (anti-HCV) 3.1% [6]. Although these data are extracted predominantly from institution-based studies and may not be representative of the situation nationwide, chronic HBV infection is thought to be a major cause of CLD in this region [4].

Community-based, longitudinal studies have been undertaken in several rural areas of Ethiopia, in recent years, using a verbal autopsy method to assign causes of death [7–9]. CLD was the leading cause of death in the age group 15–49 years in Kersa in eastern Ethiopia (13.7%) [9] and in Butajira in central Ethiopia (11.3%) [7]. In contrast, CLD was the cause of death in only 3.5% of adults of the same age in Kilte Awlalo in northern Ethiopia [8]. One suggested explanation for this difference is the relative availability of khat (*Catha edulis*), an indigenous plant which is chewed for its psychotropic effects. Khat chewing has been associated with the development of CLD [10]; its use is widespread in eastern [11] and south-central Ethiopia [12] but much less so in northern parts of the country [13].

One of the most important aspects of CLD prevention is the identification and management of potential risk factors. Public health efforts to reduce the toll of CLD in Ethiopia and other countries in sub-Saharan Africa will be considerably hampered if information on avoidable or treatable risk factors is unavailable. Thus, the aims of this study were to explore the aetiological spectrum of CLD in eastern Ethiopia and to identify plausible underlying risk factors for CLD using a hospital-based cross-sectional design.

## Methods

### Study setting and participants

A cross-sectional study of indigenous adults, aged ≥18 years, presenting for the first time with features of CLD was undertaken in two governmental hospitals in Harar, eastern Ethiopia between April 2015 and April 2016. CLD was defined as: (i) the presence of clinical features suggestive of decompensated liver disease viz. ascites, jaundice and/or hepatic encephalopathy; and (ii) the presence, on ultrasound, of hepatic parenchyma heterogeneity and/or surface irregularity. Patients presenting with severe acute hepatitis defined as liver injury of < 6 weeks duration, serum alanine aminotransferase (ALT) activity of > 100 U/L and the absence of coarsened echotexture and surface irregularity on ultrasonography, were excluded. Also excluded were patients with liver dysfunction secondary to comorbidities viz. congestive cardiac failure, biliary obstruction and septicaemia. Patients who had previously diagnosed CLD were excluded since they might represent a subgroup with more severe liver disease, or might have altered their risk habits in response to previous medical advice.

### Patient assessment

Suitable patients presenting to the regional Hiwot Fana Specialized University Hospital, and the local Jugal Hospital underwent a semi-structured interview by local nurses fluent in their mother tongue. Demographic data were recorded and potential risk factors for CLD were explored. Information on past and current use of alcohol was obtained and quantified in grams. Daily alcohol consumption of > 20 g in women and > 30 g in men, for a minimum period of 6 months, was classified as alcohol misuse. Information on khat usage was obtained using a visual analogue scale and quantified in grams. The frequency and duration of khat use in years was used to classify lifetime khat exposure as *khat-years*. Approximately 100–300 g of fresh khat leaves are chewed in a typical session [14]; thus, one khat-year was defined as daily use of 200 g of fresh khat for 1 year.

Clinical examination was undertaken using a pre-specified proforma.

### Laboratory tests

Blood was collected by venous puncture for immediate processing; serum and plasma were separated and stored in aliquots at − 20 °C until transported on ice/dry ice for analysis either in Ethiopia or Norway. Full blood counts were performed using a KX-21 N™ haematology analyser (Sysmex, Kobe, Japan). Standard biochemical tests were analysed using a semi-automatic biochemistry analyser DR-7000D (DIRUI, Changchun, China) and HumaLyzer 3000 (HUMAN, Wiesbaden, Germany). The serum aspartate aminotransferase (AST) to platelet ratio index (APRI) was calculated as $\frac{\ \frac{\mathrm{AST}\ \left(\mathrm{U}/\mathrm{L}\right)}{\mathrm{upper}\ \mathrm{reference}\ \mathrm{range}\ \mathrm{of}\ \mathrm{AST}\ \left(\mathrm{U}/\mathrm{L}\right)}\ }{\mathrm{platelet}\ \mathrm{count}\ \left({10}^9/\mathrm{L}\right)}\times 100$ [15], using a threshold of 0.7 as indicator of significant fibrosis [16]. The Fibrosis-4 (FIB-4) score was calculated as $\frac{\mathrm{age}\ \left(\mathrm{years}\right)\ \mathrm{x}\ \mathrm{AST}\ \left(\mathrm{U}/\mathrm{L}\right)}{\mathrm{platelet}\ \mathrm{count}\ \left({10}^9/\mathrm{L}\right)\ \mathrm{x}\ \sqrt{\mathrm{ALT}\ \left(\mathrm{U}/\mathrm{L}\right)}}$, using a threshold of 3.25 to indicate advanced fibrosis/cirrhosis [17].

HBsAg was measured using the rapid diagnostic test (RDT) Determine™ (Alere, Waltham, MA, USA); anti-HCV was measured using the SD BIOLINE HCV RDT (Standard Diagnostics, Yongin-si, Republic of Korea). Confirmatory testing of HBsAg and anti-HCV was undertaken using enzyme-linked immunosorbent assays (Elisys Uno, HUMAN, Wiesbaden, Germany; or Architect, Abbott Diagnostics, IL, USA). HBV DNA and HCV RNA were measured in patients who tested positive for HBsAg or anti-HCV by polymerase chain reaction using RealTime HBV, m2000 system (Abbott Molecular, Abbott Park, IL, USA). Plasma was analysed for hepatitis D virus (HDV) antigen and HDV-antibody using the ETI-DELTAK-2 and ETI-AB-DELTAK-2 assay (DiaSorin, Turin, Italy), respectively.

Human immunodeficiency virus (HIV) screening was performed using the KHB HIV (1 + 2) Antibody RDT (Shanghai Kehua Bio-Engineering, Shanghai, China) and confirmed using the HIV 1/2 STAT-PAK® RDT (Chembio Diagnostics, Medford, NY, USA). Malaria screening was performed using the SD BIOLINE Malaria Ag P.f/P.v RDT (Standard Diagnostics) and confirmed by microscopy of blood smears.

Serum was analysed for immunoglobulin G using the IMMAGE® 800 Immunochemistry System (Beckman Coulter, Brea, CA, USA). Serum iron and transferrin concentrations were quantified using ARCHITECT ci16200 (Abbott Diagnostics). Total iron binding capacity (TIBC) was calculated as 25.1 × serum transferrin (g/L) and transferrin saturation as $\frac{\mathrm{serum}\ \mathrm{iron}\ \left(\upmu \mathrm{mol}/\mathrm{L}\right)}{\mathrm{TIBC}\ \left(\upmu \mathrm{mol}/\mathrm{L}\right)}\ 100\%$.

Anti-nuclear, anti-mitochondrial and anti-actin antibodies were analysed by the Phadia™250 Laboratory system (Thermo Fisher Scientific, Waltham, MA, USA) using the EliA™ Symphony assay (Phadia, Freiburg, Germany), QUANTA Lite® M2 EP (MIT3) and QUANTA Lite® Actin IgG (Inova Diagnostics, San Diego, CA, USA).

A stool sample was collected and five thick smears processed according to a modified Kato-Katz technique using 41.7-mg templates for detection of the ova of *Schistosoma mansoni* [18].

Patients who, after initial screening, appeared to have unexplained CLD underwent more extensive testing including: measurement of serum alpha-1-antitrypsin and caeruloplasmin concentrations using the IMMAGE® 800 Immunochemistry System (Beckman Coulter); high iron Fe (*HFE*) genotyping, if the serum transferrin saturation was increased above 50%, without obvious explanation; and, screening for visceral leishmaniasis using a recombinant K39-antigen strip test IT-LEISH® (Bio-Rad) and confirmed by Giemsa stained splenic smear.

Urine from all women < 45 years of age was tested for human chorionic gonadotropin (hCG) using a HCG Pregnancy Strip Test (Nantong Egens Biotechnology, Jiangsu, China).

### Abdominal imaging

Abdominal ultrasonography was undertaken to a pre-determined standard by a local radiologist using a 3.5 MHz convex transducer Flexus SSD-1100 (Aloka, Tokyo, Japan). The diagnosis of CLD was based on the presence of an irregular liver surface and/or liver parenchyma heterogeneity [19]. The presence of schistosomal periportal fibrosis was diagnosed using WHO criteria [20] and re-evaluated by an independent expert.

### Determination of the aetiology of the CLD

Historical, clinical, laboratory and imaging data were used to identify the aetiology of the underlying CLD using published criteria (Table 1) [21–25].

**Table 1:** Criteria used to assign the aetiology of the liver disease

Aetiology		Criteria used to assign diagnosis
1	Chronic hepatitis B infection	Evidence of CLD on liver ultrasound and positive serum HBsAg.
2	Chronic hepatitis C infection	Evidence of CLD on liver ultrasound and positive serum anti-HCV and positive HCV RNA.
3	Chronic hepatitis D infection	Chronic hepatitis B infection and positive serum anti HDV IgG confirmed by detection of HDV RNA.
4	Primary biliary cholangitis	i. Strongly positive anti-mitochondrial antibodies and ii. Cholestatic liver function tests: a. ALP > 1.5 x URR and b. AST < 5 x URR
5	Autoimmune hepatitis^a^	i. Strongly positive anti-nuclear antibodies or anti-actin and ii. Elevated IgG > 1.1 x URR
6	Alcoholic liver disease	i. Clinical and radiological signs of CLD and ii. Daily alcohol consumption > 20 g/day in women and > 30 g/day in men for 6 months or more.
7	Non-alcoholic fatty liver disease	i. Liver ultrasound findings of steatosis and ii. Absence of significant alcohol consumption^b^ or other recognised secondary causes of steatosis and iii. BMI > 25 kg/m^2 c^
8	Haemochromatosis	i. Transferrin saturation > 50% and ii. Genotyping showing C282Y homozygosity or C282Y/H63D heterozygosity or C282Y/S65C heterozygosity on the HFE gene.
9	Wilson’s disease	i. Serum caeruloplasmin < 0.140 g/L and ii. Age < 40 years
10	Alpha-1-antitrypsin deficiency	Serum alpha-1-antitrypsin level < 0.85 g/L.
11	Malaria	Positive malaria rapid diagnostic test and positive microscopy.
12	Hepatic schistosomiasis	Presence of ova from *Schistosoma mansoni* in Kato-Katz thick stool smears and typical liver ultrasound findings viz. periportal thickening/‘pipestem’ fibrosis confirmed by an independent expert.
13	Visceral leishmaniasis	Ultrasound findings of hepatosplenomegaly and positive K39 antigen strip test confirmed by positive splenic smear.
14	Unexplained chronic liver disease	None of the above

*Abbreviations*: *ALP* alkaline phosphatase, *anti-HCV* hepatitis C virus antibody, *anti-HDV* hepatitis D virus antibody, *AST* aspartate aminotransferase, *BMI* body mass index, *CLD* chronic liver disease, *HBsAg* hepatitis B surface antigen, *HCV* hepatitis C virus, *HDV* hepatitis D virus, *HFE* high iron Fe, *IgG* immunoglobulin G, *URR* upper reference range

Laboratory reference ranges: ALP (60–306 U/L); AST (14–40 U/L); IgG (0.8–27.8 g/L) [21]

^a^Based on the American Association for the Study of Liver Disease (AASLD) simplified criteria [22] in the absence of histology

^b^Alcohol consumption < 20 g/day in women and < 30 g/day in men

^c^Not a part of the AASLD criteria [23] but adopted to exclude cases of starvation-induced steatosis

### Liver biopsy and histopathology

It was intended that all patients in whom the aetiology of the CLD remained unexplained following investigation would be offered a liver biopsy. However, during the period April 2015 to April 2016, no suitably trained personnel were available to undertake this procedure. This situation was eventually resolved and the patients were subsequently contacted and asked to return for liver biopsy. In the interim several of the more decompensated patients had died and as the biopsies were to be performed percutaneously, only those with a normal or marginally elevated prothrombin time were considered suitable [26].

The procedure was performed, under ultrasound guidance, using a sterile Menghini technique with local anaesthetic and a 17G needle Hepafix® (Braun, Melsungen, Germany). Serial four μm sections were cut and stained with haematoxylin and eosin; Gomori (reticulin); van Gieson (collagen); Masson Trichrome (metachromatic); periodic acid-Schiff (PAS), with and without diastase (glycogen); and Perls (iron). Histopathologists in Norway and London independently assessed the histological findings blinded to the clinical information; inflammation and fibrosis were graded and staged using the semi-quantitative, modified Histological Activity Index [27]. Subsequent immunohistochemistry was undertaken using Ki-67 as a proliferation marker (Dako, catalogue number M724, concentration 1/100 with pre-treatment) and activated caspase-3, (Cell Signalling Technology, catalogue number 9664, concentration 1/100 with pre-treatment) as an apoptotic marker. Image analysis to quantify the degree of fibrosis and to calculate the collagen proportionate area (CPA) was carried out on scanned, Sirius Red stained sections [28].

### Statistical methods

Statistical analyses were performed in SPSS 23.0 (SPSS Inc., Chicago, IL, USA). Categorical variables were summarized as frequencies, while continuous variables were presented as median and interquartile range (IQR). Comparisons between groups were performed using the Pearson χ^2^-test for categorical variables and Mann-Whitney U-test for continuous variables. A *p*-value < 0.05 was considered significant. The *Strengthening the Reporting of Observational studies in Epidemiology* (STROBE) statement guidelines were followed [29].

### Ethics

The study was approved by the National Research Ethics Review Committee (Ref. No.: 3.10/829/07 and 3.10/129/2016) in Ethiopia and by the Regional Committees for Medical and Health Research Ethics (Ref. No.: 2014/1146) in Norway. The study was conducted in accordance with the Declaration of Helsinki [30]. Written informed consent was obtained from all participating individuals.

## Results

### Study population

**Fig. 1:**
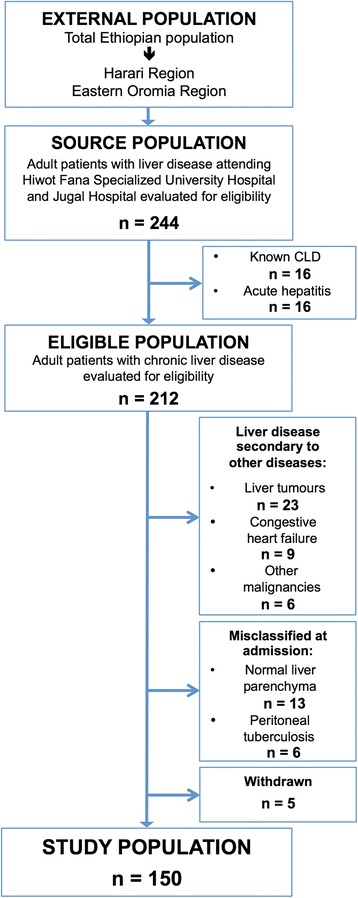
Study flow diagram illustrating the selection of the study subjects. Abbreviations: CLD, chronic liver disease

A total of 244 patients with liver disease were admitted to hospital during the study period. Of these, 212 patients presented with a new diagnosis of probable CLD and were evaluated for inclusion. The final study population comprised of 150 cases with newly diagnosed CLD (Fig. 1).

### Aetiological spectrum

The aetiology of the liver disease was identified in 67 (44.7%) of the 150 patients and ascribed to chronic HBV infection in 55 (36.7%); hepatic schistosomiasis in four (2.7%); alcohol misuse in three (2.0%); chronic HCV infection in two (1.3%); autoimmune hepatitis in two (1.3%) and visceral leishmaniasis in one (0.7%). No cause was identified in the remaining 83 (55.3%) patients, in whom the liver disease was, therefore, unexplained.

### Demography

Overall, there were more men (72.0%) than women; the median age was 30 (IQR 25–40) years (Table 2). The majority of the study subjects were Muslim (92.7%). The overall reported prevalence of daily khat use was 78.0%. Khat use was more common among men than women (92.6% vs. 40.5%; *p* < 0.001); overall khat exposure was also higher in men than women (36 vs. 0.6 khat-years; *p* < 0.001).

**Table 2:** Demographic features of the study subjects with chronic liver disease, by aetiology

Variable	All patients (*n* = 150)	Aetiology known (*n* = 67)	Aetiology unknown(*n* = 83)
Sex (n, % men)	108 (72.0)	55 (82.1)	53 (63.9)^*^
Age (years)	30 (25–40)	30 (20–40)	30 (25–40)
Ethnic group			
Oromo	134 (89.3)	59 (88.1)	75 (90.4)
Amhara	9 (6.0)	5 (7.5)	4 (4.8)
Somali	5 (3.3)	2 (3.0)	3 (3.6)
Gurage	2 (1.3)	1 (1.5)	1 (1.2)
Religion			
Islam	139 (92.7)	60 (89.6)	79 (95.2)
Christianity	11 (7.3)	7 (10.4)	4 (4.8)
Occupation			
Farmer	100 (66.7)	46 (68.7)	54 (65.1)
Unemployed	14 (9.3)	5 (7.5)	9 (10.8)
Housewife	11 (7.3)	2 (3.0)	9 (10.8)
Student	8 (5.3)	5 (7.5)	3 (3.6)
Day worker	5 (3.3)	3 (4.5)	2 (2.4)
Public servant	4 (2.7)	1 (1.5)	3 (3.6)
Health professional	2 (1.3)	2 (3.0)	0
Other	6 (4.0)	3 (4.5)	3 (3.6)
Pregnant	3 (2.0)	1 (8.3)	2 (6.7)
Previous blood transfusion	23 (15.3)	9 (13.4)	14 (16.9)
Family history of liver disease	8 (5.3)	4 (6.0)	4 (4.8)
Dietary grain stored underground	53 (35.3)	25 (37.3)	28 (33.7)
Weeks of storage	24 (12–52)	24 (12–52)	24 (12–52)
Traditional herbal medicine	40 (26.7)	16 (23.9)	24 (28.9)
History of alcohol consumption:			
Never	139 (92.7)	61 (91.0)	78 (94.0)
Current	6 (4.0)	5 (7.5)	1 (1.2)
Stopped	5 (3.3)	1 (1.5)	4 (4.8)
Alcohol abuse^a^	3 (2.0)	3 (4.5)	0
History of daily use of khat	117 (78.0)	56 (83.6)	61 (73.5)
Khat-years^b^	20 (3–70)	20 (3–75)	18 (1–60)

Data are presented as number (%) or as median (interquartile range) unless otherwise noted

^*^p < 0.05; significance of the difference between the aetiology known/unknown group

^a^Daily consumption of > 20 g/day in women and > 30 g/day in men for 6 months or more

^b^One khat-year is defined as daily use of 200 g fresh khat for 1 year

Women were more likely to have unexplained CLD than men (71.4% vs. 49.1%; *p* = 0.013). Otherwise there were no significant differences in demographic features between the aetiology known/unknown groups.

### Clinical presentation

The majority of the patients presented with clinical features suggestive of hepatic decompensation (Table 3). Patients with unexplained CLD were more likely to present with abdominal swelling than those in whom the aetiology was known (92.8% vs. 76.1%; *p* = 0.004). Otherwise there were no distinguishing clinical features between the aetiology known/unknown groups.

**Table 3:** Clinical characteristics and ultrasound findings in the study subjects with chronic liver disease, by aetiology

Variable	All patients (*n* = 150)	Aetiology known (*n* = 67)	Aetiology unknown (*n* = 83)
Symptoms			
Abdominal swelling	128 (85.3)	51 (76.1)	77 (92.8)^*^
Epigastric pain	12 (80.0)	56 (83.6)	64 (77.1)
Weight loss	119 (79.3)	54 (80.6)	65 (78.3)
Fever	77 (51.3)	35 (52.2)	42 (50.6)
Arthralgia/myalgia	75 (50.3)^a^	33 (49.3)	42 (51.2)^a^
Nausea	69 (46.3)^a^	30 (45.5)^a^	39 (47.0)
Diarrhoea	64 (42.7)	27 (40.3)	37 (44.6)
Haematemesis	53 (35.3)	27 (40.3)	26 (31.3)
History of jaundice	47 (31.3)	24 (35.8)	23 (27.7)
Clinical findings			
Ascites	138 (92.0)	60 (89.6)	78 (94.0)
Splenomegaly	99 (66.0)	48 (71.6)	51 (61.4)
Jaundice	28 (18.7)	16 (23.9)	12 (14.5)
Caput medusae	25 (16.7)	8 (11.9)	17 (20.5)
Hepatic encephalopathy	16 (10.7)	7 (10.4)	9 (10.8)
Traditional scarring/burning	101 (67.3)	46 (68.7)	55 (66.3)
Ultrasound findings			
Ascites	138 (92.0)	60 (89.6)	78 (94.0)
Smooth liver surface	4 (2.7)	3 (4.5)	1 (1.2)
Mild uneven liver surface	44 (29.3)	15 (22.4)	29 (34.9)
Nodular liver surface	102 (68.0)	49 (73.1)	53 (63.9)
Heterogeneous echotexture	62 (41.3)	22 (32.8)	40 (48.2)
Coarse echotexture	87 (58.0)	44 (65.7)	43 (51.8)
Hepatic steatosis	1 (0.7)	1 (1.5)	0
Periportal fibrosis	21 (14.0)	9 (13.4)	12 (14.5)
In-hospital death	9 (6.0)^a^	4 (6.0)	5 (6.1)^a^

Data are presented as number (%) or as median (interquartile range) unless otherwise noted

^a^One observation missing

^*^p < 0.05; significance of the difference between the aetiology known/unknown group

### Laboratory findings

Overall, the alterations in laboratory variables were mild (Table 4). A total of 92 (61.7%) patients had an APRI score of > 0.7 while 43 (28.9%) had a FIB-4 score of > 3.25. Patients with unexplained CLD had a lower median serum ALT activity (30 U/L [IQR 21–51] vs. 41 [IQR 24–58]; *p* = 0.032) and values above the upper reference range (URR) were observed in proportionately fewer patients than amongst those in whom the aetiology was known (31.3% vs. 50.7%; *p* = 0.016). Otherwise there were no distinguishing laboratory features between the aetiology known/unknown groups.

**Table 4:** Laboratory findings in the study subjects with chronic liver disease, by aetiology

Laboratory variable	All patients (*n* = 150)	Aetiology known (*n* = 67)	Aetiology unknown (*n* = 83)
ALT (U/L)	34 (22–55)	41 (24–58)	30 (21–51)^*^
> URR	60 (40.0)	34 (50.7)	26 (31.3)^*^
AST (U/L)	44 (28–81)	52 (31–83)	41 (28–78)
> URR	84 (56.0)	41 (61.2)	43 (51.8)
ALP (U/L)	317 (207–416)	315 (250–423)	320 (200–385)
> URR	80 (53.3)	37 (55.2)	43 (51.8)
GGT (U/L)	29 (19–48)	29 (21–47)	29 (18–52)
> URR	30 (20.0)	14 (20.9)	16 (19.3)
Total bilirubin (μmol/L)	19 (10–38)	21 (12–51)	17 (10–31)
> URR	36 (24.0)	20 (29.9)	16 (19.3)
Albumin (g/L)	37 (28–50)	36 (30–50)	37 (27–50)
< LRR	63 (42.0)	27 (40.3)	36 (43.4)
Creatinine (μmol/L)	80 (62–97)	80 (62–97)	71 (62–88)
> URR	23 (15.3)	11 (16.4)	12 (14.5)
< LRR	19 (12.7)	8 (11.9)	11 (13.3)
Platelet count (10^9^/L)	125 (76–206)	123 (71–186)	147 (76–223)
< LRR	75 (50.0)	36 (53.7)	39 (47.0)
IgG (g/L)	23.9 (17.1–32.5)	27.0 (16.7–34.2)	21.6 (17.2–30.6)
> URR	55 (36.7)	31 (46.3)	24 (28.9)
HIV infection^a^	4 (2.7)	1 (1.5)	3 (3.6)
Kato-Katz smear positive	23 (16.5)^b^	13 (21.3)^c^	10 (12.8)^d^
APRI score > 0.7^e^	92 (61.7)^f^	46 (69.7)^f^	46 (55.4)
FIB-4 score > 3.25^g^	43 (28.9)^f^	20 (30.3)^f^	23 (27.7)
APRI score > 0.7 OR FIB-4 score > 3.25	94 (63.1)^f^	46 (69.7)^f^	48 (57.8)

Data are presented as number (%) or as median (interquartile range)

Laboratory reference ranges: ALT (8–40 U/L); AST (14–40 U/L); ALP (60–306 U/L); GGT (7–61 U/L); Bilirubin (3–38 μmol/L); Albumin (35–52 g/L); Creatinine (47–109 μmol/L); Platelet count (126–438 × 10^9^/L); IgG (0.8–27.8 g/L) [21]

^a^One patient with chronic HCV was co-infected with HIV.

^b^Stool sample missing in 11 patients.

^c^Stool sample missing in six patients.

^d^Stool sample missing in five patients.

^e^APRI: (AST (U/L)/URR of AST (U/L))/platelet count (10^9^/L) × 100

^f^One observation missing.

^g^FIB-4: age (years) x AST (U/L)/(platelet count (10^9^/L) x √ALT (U/L))

*p < 0.05; significance of the difference between the aetiology known/unknown group

*Abbreviations*: *ALP* alkaline phosphatase, *ALT* alanine aminotransferase, *APRI* aspartate aminotransferase to platelets ratio index, *AST* aspartate aminotransferase, *GGT* gamma-glutamyltransferase, *HCV* hepatitis C virus, *HIV* human immunodeficiency virus, *IgG* immunoglobulin G, *LRR* lower reference range, *URR* upper reference range

### Abdominal ultrasound findings

The commonest findings on liver ultrasound were an irregular/nodular liver surface (68.0%), coarse liver texture (58.0%) and ascites (92.0%) (Table 3). There were no significant differences in abdominal ultrasound findings between the aetiology known/unknown groups.

### Histopathology

Of the 83 patients with unexplained CLD, 15 (18.1%) died during or shortly after admission; 35 (42.2%) could not be contacted; four (4.8%) refused to undergo the procedure, while 24 (28.9%) were unsuitable because of a severe coagulopathy. Thus, only five (6.0%) patients underwent liver biopsy a median (range) of 33 (20–64) weeks after their initial hospitalization (Table 5); three (Cases 1/2/4) had a history of khat chewing.

**Table 5:** Characteristics of the five patients with unexplained chronic liver disease who underwent liver biopsy

No.	Sex	Age (yr)	Alcohol use	Khat use	Main symptoms and signs	Main ultrasound findings	ALT (U/L)	AST (U/L)	ALP (U/L)	Albumin (g/L)	Bilirubin (μmol/L)	APRI score^a^	FIB-4 score^b^	Biopsy delay (wk)
1	Female	26	Never	100 g/day, 10 yr	Abdominal swelling; abdominal pain; nausea; diarrhoea; fatigue Ascites	Heterogenous liver texture Mild uneven liver surface Gross ascites Moderate splenomegaly	15	16	88	50	9	0.20	0.53	20
2	Male	25	Never	200 g/day, 5 yr	Abdominal pain; Diarrhea; arthralgia / myalgia	Heterogenous liver texture Mild uneven liver surface Periportal fibrosis Moderate splenomegaly	15	19	107	46	19	0.74	1.92	33
3	Female	30	Never	Never	Abdominal swelling; abdominal pain Ascites; umbilical hernia; non-specific rash	Heterogenous liver texture Mild uneven liver surface Gross ascites Reduced liver span	43	44	258	20	5	0.68	1.25	64
4	Male	25	36 g/day × 1/wk., 3 yr	400 g/day × 3/wk., 5 yr	Abdominal pain; nausea; diarrhea; arthralgia/myalgia Splenomegaly	Heterogenous liver texture Mild uneven liver surface Splenomegaly	100	98	300	58	31	1.34	1.34	22
5	Female	25	Never	Never	Abdominal swelling; fatigue; peripheral oedema. Ascites	Heterogenous liver texture Mild uneven liver surface Gross ascites Reduced liver span	39	62	385	27	17	0.76	1.22	58

*Abbreviations*: *ALP* alkaline phosphatase, *ALT* alanine aminotransferase, *APRI* aspartate aminotransferase to platelets ratio index, *AST* aspartate aminotransferase, *URR* upper reference range

Laboratory reference ranges: ALT (8–40 U/L); AST (14–40 U/L); ALP (60–306 U/L); Albumin (35–52 g/L); Bilirubin (3–38 μmol/L) [21]

^a^APRI: (AST (U/L)/URR of AST (U/L))/platelet count (10^9^/L) × 100

^b^FIB-4: age (years) x AST (U/L)/(platelet count (10^9^/L) x √ALT (U/L))

**Fig. 2:**
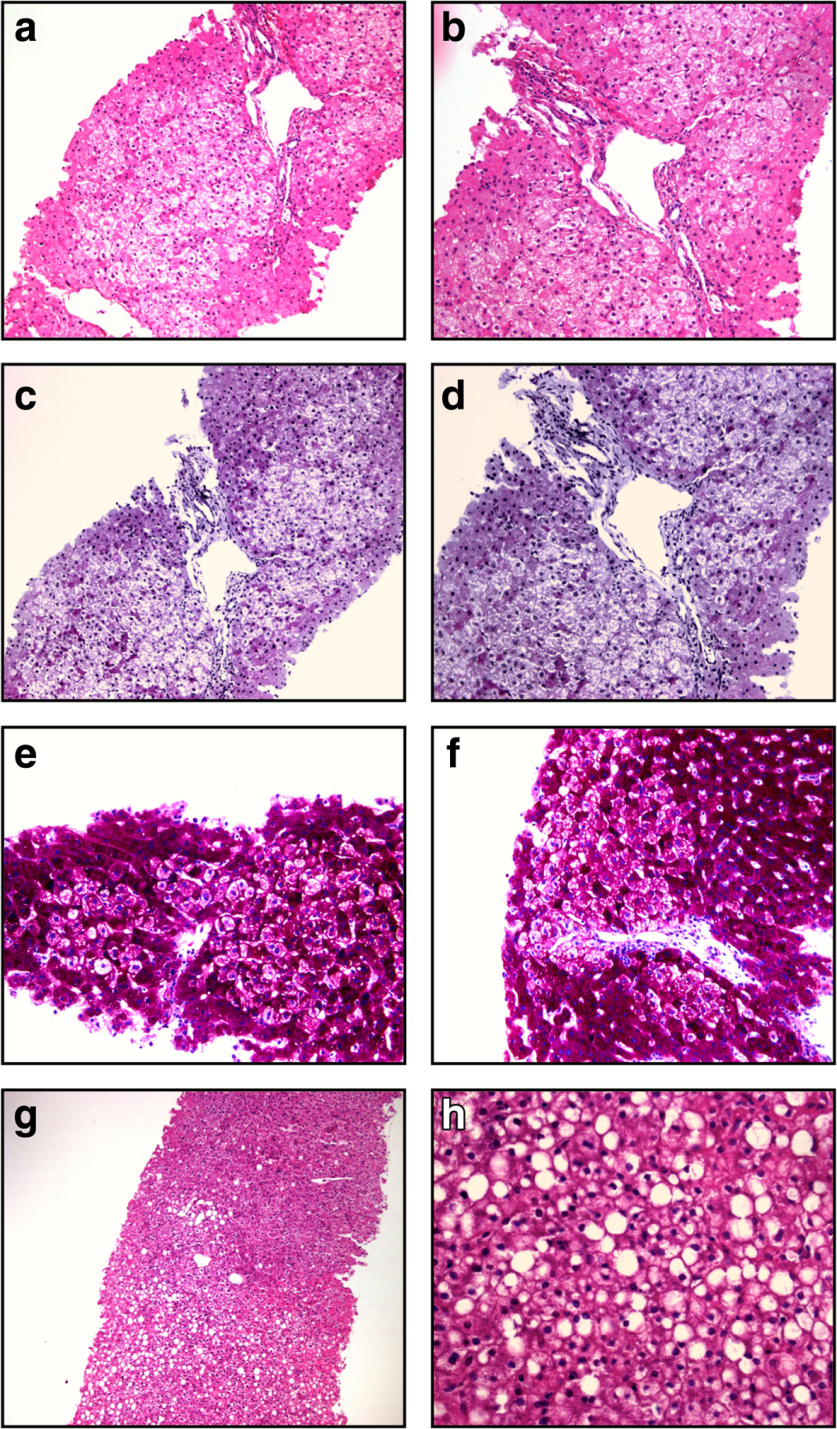
Liver histology in Ethiopian patients with unexplained chronic liver disease. **a** Case 1: Adaptive parenchymal changes with focal diffuse swollen pale stained hepatocytes stretching through all zones. H&E, 100×. **b** Case 1: Intact liver plate, normal bile ducts and patent central vein. Sinusoidal lymphocytosis. H&E, 200×. **c** + **d** Case 1: Masson Trichrome stain negative indicated non-cirrhotic liver disease. 100×, 200×. **e** + **f** Case 1: Swollen hepatocyte clarification negative for periodic acid-Schiff stain in approximately 30%. 200×. **g** Case 5: Mild mixed steatosis. H&E, 100×. **h** Case 5: Mixed macro- and microvesicular steatosis with focal single cell necrosis and sporadic parenchymal granulocytes. H&E, 400×. Abbreviations: H&E, haematoxylin and eosin

Microscopically none of the specimens showed more than mild fibrosis and inflammation (Table 6). Foci of pale stained swollen hepatocytes were identified in Cases 1–4, with no marked zonal distribution; these stained negative for PAS and were thus suggestive of toxic injury (Case 1; Fig. 2a-f). The fifth patient showed no evidence of adaptive parenchymal changes but mild mixed steatosis and focal single cell necrosis (Fig. 2g-h). Although the numbers were modest and the differences small, the proliferation index, apoptotic scores and the CPA tended to be higher among the three patients (Cases 1/2/4) who chewed khat compared to the two who did not (Cases 3/5).

**Table 6:** Histopathological findings of the five patients with unexplained chronic liver disease who underwent liver biopsy

No	Sex	Age (yr)	General microscopy	Parenchymal changes	Ishak-score
1	Female	26	Mild portal and lobular hepatitis with sinusoidal lymphocytosis. Variation of hepatic cord thickness. Normal bile ducts and intact liver plate.	Focal adaptive parenchymal changes with diffuse hepatocyte swelling/ clarification. No steatosis or haemosiderosis. Collagen proportionate area: 5% Proliferation index: 8% Apoptosis index: 3%	Fibrosis = 2 Necroinflammation = 2 • piecemeal 0 • lobular 1 • confluent 0 • portal 1
2	Male	25	Normal architecture with normal portal areas, no inflammation. Normal bile ducts and intact liver plate.	Adaptive parenchymal changes with diffuse hepatocyte swelling/ clarification. No steatosis or haemosiderosis. Collagen proportionate area: 6% Proliferation index: 12% Apoptosis index: 5%	Fibrosis = 1 Necroinflammation = 0
3	Female	30	Normal architecture with normal portal areas, no inflammation. Normal bile ducts and intact liver plate.	Mild adaptive parenchymal changes with diffuse hepatocyte swelling/clarification. No steatosis or haemosiderosis. Collagen proportionate area: 3% Proliferation index: 6% Apoptosis index: 1%	Fibrosis = 1 Necroinflammation = 1 • piecemeal 0 • lobular 1 • confluent 0 • portal 0
4	Male	25	Normal architecture with normal portal areas, no inflammation. Normal bile ducts and intact liver plate.	Adaptive parenchymal changes with diffuse hepatocyte swelling/clarification. No steatosis or haemosiderosis. Collagen proportionate area: 8% Proliferation index: 15% Apoptosis index: 6%	Fibrosis = 0 Necroinflammation = 0
5	Female	25	Normal architecture with normal portal areas. Normal bile ducts and intact liver plate	Mild mixed steatosis ≈ 20% Focal single cell necrosis, a few apoptotic hepatocytes, a few parenchymal granulocytes. No adaptive changes. Collagen proportionate area: 3% Proliferation index: 3% Apoptosis index: 2%	Fibrosis = 0 Necroinflammation = 1 • piecemeal 0 • lobular 1 • confluent 0 • portal 0

## Discussion

This study aimed to explore the aetiological spectrum and underlying risk factors for the development of CLD in eastern Ethiopia. Chronic HBV infection was the major identified risk factor, explaining the development of CLD in roughly one-third of the patients. However, an aetiological factor was identified in less than 10% of the remainder. Thus, in over half of the included cases the aetiology of the liver disease was unexplained.

Of prime importance in this study was the surety of the diagnosis of CLD. The criteria used were stringent and required not only that patients had clinical evidence of decompensated liver disease but also evidence of hepatic parenchyma heterogeneity and/or surface irregularity on ultrasound. The liver function test abnormalities were mild but this is not incompatible with the diagnosis of CLD. Over two-thirds of the patients had APRI and/or FIB-4 scores compatible with a diagnosis of significant fibrosis/cirrhosis. The histological findings in the five patients who underwent liver biopsy would seem at odds with a diagnosis of CLD; however, these patients fulfilled the inclusion criteria at presentation and biopsies undertaken after a considerable delay still showed evidence of ongoing disease. Thus, the fact that the patients included in this study had CLD can be accepted with a high degree of certainty.

The proportion of patients, in the present study, in whom the CLD was aetiologically unexplained is substantially higher than might be expected. In the 1980’s more than 50% of cases of CLD worldwide did not have an ascribed cause compared with the current global estimate of approximately 5% [31–33]. Thus, the prevalence of unexplained CLD in this area of eastern Ethiopia is ten-fold higher than would be expected. No observational studies exploring the aetiological spectrum of CLD in eastern Ethiopia or in sub-Saharan Africa are available for comparison.

The seroprevalence of HBsAg in the present population was high while the seroprevalence of anti-HCV was low. There are no representative population-based prevalence studies on viral hepatitis in this part of Ethiopia. However, a recent study of blood donors in eastern Ethiopia found similar seroprevalence rates to those reported here [34].

No data are available on the prevalence of NAFLD in Ethiopia although it is known that Ethiopia has one of the lowest prevalence rates of obesity worldwide [35]. The data that are available from other populations suggest that the overall prevalence of NAFLD in sub-Saharan Africa is low [36]. In a case-control study undertaken in Nigeria, 16.7% of patients with type II diabetes mellitus were found to have NAFLD compared with only 1.2% of non-diabetic control subjects, suggesting that, in comparison with Caucasian, Indian and Asian populations, diabetes may be a more important risk factor for NAFLD in Africa than obesity [37]. None of the patients in the present study was obese; other than one case with alcoholic liver disease, none had significant steatosis on hepatic ultrasound and only one had diabetes. Thus, the prevalence of NAFLD in this study population is likely to be very low.

The prevalence of daily khat use identified in the present study was much higher than previously reported [11, 13]. A regional study in Harar city found that 20.9% of 1890 secondary school students chewed khat daily; the lifetime prevalence of khat chewing was 24.2% [11]. The 2011 Ethiopian Demographic and Health Survey identified an overall prevalence of khat chewing of 15.3%. However, there are significant regional variations in the prevalence from 53.2% in the Harari region in eastern Ethiopia to 1.1% in the Tigray region in northern Ethiopia [13]. Khat use is more widespread amongst Muslims than Christians and amongst men than in women [11, 13], which accords with the findings in the present study.

There are a number of case reports which implicate khat as a factor in the development of both acute [38] and chronic liver disease [39–41]. In addition, khat-related hepatotoxicity has been convincingly demonstrated in animal models [42]. The fact that khat use was similar amongst patients with and without other risk factors indicated that it may act as a sole or an adjuvant cause of liver injury. Although only a limited number of liver biopsies was undertaken, the histological findings of focal parenchymal changes mirror those observed in animal models [42] and are supportive of toxic liver injury. However, the design of this study does not allow a definitive conclusion to be made, and further studies to assess causality are needed.

This study had a number of strengths despite the resource limitations at the Ethiopian sites. First: the sample size was large and the prospective inclusion of study subjects provided consistent data sampling throughout the study period. Second: robust clinical, laboratory and ultrasound criteria were used to define CLD. Third: the aetiology of the liver injury was determined following a comprehensive, standardized clinical evaluation, multicentre laboratory testing using high-performance diagnostics, abdominal ultrasound with expert review, and, in a small number, histological examination of liver biopsy material.

The study also has its limitations. First: selection bias cannot be excluded, as an unknown proportion of patients with CLD may not have been seen by the recruiting medical services for a variety of practical, cultural and socioeconomic reasons. Second: liver biopsies were undertaken in only a small number of patients with unexplained CLD; the selection procedure for liver biopsy undoubtedly favoured those with the mildest disease and the time interval between presentation and the procedure was sufficiently long for there to have been some resolution of the liver disease. Nevertheless, the histological findings provided useful confirmatory evidence of toxic liver injury in some. Third: issue could be taken with the criteria used to diagnose schistosomal liver disease. Positive assignment required a positive stool smear and radiological evidence of periportal thickening/‘pipe stem’ fibrosis confirmed by expert opinion; thus, the diagnosis may have been underestimated. Fourth: HBV DNA levels were not measured in 95 HBsAg-negative patients and thus the presence of occult HBV could not be ruled out in this subgroup [43]. However, the pathogenetic mechanism of occult HBV infection is still not clear [44] and the role of occult HBV in unexplained CLD is still debated [33]. Approximately 95% of the patients with unexplained CLD in the present study had decompensated disease on presentation but only low-grade abnormalities in the liver transaminase activities. Thus, it is unlikely that occult HBV infection was the underlying cause of the unexplained CLD in this population. Finally: the diagnosis of CLD was not confirmed by advanced imaging, endoscopy or, in the majority, by histological examination of liver biopsy material. Furthermore, certain causes of CLD could not be ruled out due to resource limitations, including: primary sclerosing cholangitis, veno-occlusive disease/Budd-Chiari syndrome and injury from other hepatotoxins.

CLD has recently been reported as the leading cause of death in adults less than 50 years of age in eastern Ethiopia [9]. If, as identified in the present study, a high proportion of the CLD is ‘unexplained’ then it may be difficult, if not impossible, to prevent its occurrence and hence to reduce the burden it imposes. If, however, as suggested in the present study, exposure to the recreational substance khat is of major aetiological importance, then there is an urgent need to further investigate this possibility with analytic studies designed to assess causality. There are campaigns in place to radically reduce the burden of viral liver disease worldwide [45], and this is undoubtedly vital. However, if khat was found to be a major contributor to the development of CLD, then given its widespread use, legal status and social acceptability it would be a much more difficult problem to deal with requiring concerted governmental action in the countries and communities involved.

## Conclusions

Chronic HBV infection was found in around one third of patients hospitalized with CLD in eastern Ethiopia. However, in over half of the patients the aetiology of the liver disease was unexplained. The prevalence of khat chewing was much higher in the CLD population than expected, suggesting khat as an effect modifier and/or independent risk factor for development of CLD in this part of the world. Further epidemiological studies, which include appropriate comparison groups, should be undertaken to assess whether khat plays a causal role in the development of CLD.

## Abbreviations

ALT: Alanine aminotransferase
anti-HCV: Hepatitis C virus antibody
anti-HDV: Hepatitis D virus antibody
APRI: Aspartate aminotransferase to platelet ratio index
AST: Aspartate aminotransferase
CLD: Chronic liver disease
CPA: Collagen proportionate area
HBsAg: Hepatitis B surface antigen
HBV: Hepatitis B virus
HCV: Hepatitis C virus
HDV: Hepatitis D virus
NAFLD: Non-alcoholic fatty liver disease
